# Forest aboveground biomass estimation based on spaceborne LiDAR combining machine learning model and geostatistical method

**DOI:** 10.3389/fpls.2024.1428268

**Published:** 2024-12-11

**Authors:** Li Xu, Jinge Yu, Qingtai Shu, Shaolong Luo, Wenwu Zhou, Dandan Duan

**Affiliations:** ^1^ Faculty of College of Soil and Water Conservation, Southwest Forestry University, Kunming, China; ^2^ Information Technology Research Center, Beijing Academy of Agriculture and Forestry Sciences, Beijing, China

**Keywords:** GEDI, spaceborne LiDAR, inverse distance weighting, biomass, spruce-fir

## Abstract

Estimation of forest biomass at regional scale based on GEDI spaceborne LiDAR data is of great significance for forest quality assessment and carbon cycle. To solve the problem of discontinuous data of GEDI footprints, this study mapped different echo indexes in the footprints to the surface by inverse distance weighted interpolation method, and verified the influence of different number of footprints on the interpolation results. Random forest algorithm was chosen to estimate the spruce-fir biomass combined with the parameters provided by GEDI and 138 spruce-fir sample plots in Shangri-La. The results show that: (1) By extracting different numbers of GEDI footprints and visualize it, the study revealed that a higher number of footprints correlates with a denser distribution and a more pronounced stripe phenomenon. (2) The prediction accuracy improves as the number of GEDI footprints decreases. The group with the highest R^2^, lowest RMSE and lowest MAE was the footprint extracted every 100 shots, and the footprint extracted every 10 shots had the worst prediction effect. (3) The biomass of spruce-fir inverted by random forest ranged from 51.33 t/hm^2^ to 179.83 t/hm^2^, with an average of 101.98 t/hm^2^. The total value was 3035.29 × 10^4^ t/hm^2^. This study shows that the number and distribution of GEDI footprints will have a certain impact on the interpolation mapping to the surface information and presents a methodological reference for selecting the appropriate number of GEDI footprints to derive various vertical structure parameters of forest ecosystems.

## 1 Introduction

As the energy base and material source of forest ecosystem operation, forest aboveground biomass (AGB) is a key indicator to evaluate the health status of forest ecosystem and the sustainable utilization of vegetation resources (Assiri et al., 2023). It is also the basis for the study of ecosystem carbon cycle and carbon storage. Spruce-fir forest is the most widely distributed forest type in Chinese cold-temperate evergreen coniferous forest, which has high economic and ecological value (Liu et al., 2023a). Spruce-fir has important ecological functions in water retention and soil and water conservation. It can not only maintain biodiversity in high altitude and high latitude areas, but also affect the sustainable development of social economy (Chen, 2023). Chinese spruce-fir resources are extremely rich, with a stand volume of 2.2 billion m^3^, accounting for about 24.23% of the national stand volume, and ranking first among all tree species (Huang et al., 2004). The spruce-fir ecosystem is one of the most productive ecosystems on earth, and its carbon storage capacity is outstanding. Because of its important carbon storage capacity, it plays an important role in mitigating climate change (Lang et al., 2019). Therefore, accurate and large-scale monitoring of the AGB of the Shangri-La spruce-fir is of great significance for ensuring the safety of the alpine ecosystem and maintaining its carbon balance. Remote sensing technology has the potential to quickly obtain large-scale vegetation growth conditions (Yun et al., 2024). However, accurate regional AGB data still requires detailed field surveys on a finer scale (Liu et al., 2023b). Combining field survey data with RS technology has become a common method for estimating regional AGB. According to the different ways of obtaining forest vertical structure and sensors, remote sensing data can be divided into optical, synthetic aperture radar (SAR) and laser radar data. At present, medium-resolution optical data is still the most commonly used data source for large-scale acquisition of forest AGB, and its rich spectral information can effectively reflect the growth of vegetation (Jiang et al., 2021). Landsat data can provide multi-spectral images with medium resolution for decades, which is the most widely used data for forest researchers to solve various practical tasks. However, cloud cover and the saturation effect of dense forests are its main limitations (Wang et al., 2021). SAR obtains information by actively emitting energy, also known as active remote sensing. Its wavelength can penetrate the vegetation canopy and obtain more detailed structural information. It has obvious advantages in obtaining the vertical structure of forests (Cartus et al., 2012). However, SAR must work in a specific range of electromagnetic spectrum, but the characteristics of these bands are not necessarily suitable for biomass estimation.

As early as the mid-1980s, LiDAR technology has been applied in the field of forestry. As an active remote sensing technology, LiDAR has strong penetrability and can overcome the signal saturation problem in SAR and optical remote sensing data (Gao et al., 2022). According to it carrying platform, it can be divided into Terrestrial Laser scanner, Airborne Laser Scanner and Space-borne Laser. Terrestrial Laser scanner is usually used for the acquisition of single target or small-scale fine 3D data (Comesaña-Cebral et al., 2021). Its top-down working model allows individual trees to be segmented to provide accurate estimates of DBH and tree position. However, due to its top-down scanning method, the information at the top of the canopy may be missing, and it is limited by terrain, scanning field of view and distance, so it is not suitable for inverting continuous large-scale forest vertical structure parameters. Airborne LiDAR is the best choice for forest AGB estimation at single tree scale due to its low cost, flexible operation and centimeter-level spatial image resolution. In recent years, it has been more and more widely used to estimate forest canopy height and biomass. However, the point cloud data of airborne LiDAR has the characteristics of large density and inhomogeneous distribution, which increases the difficulty of data processing, and the application in large areas and the acquisition of data are limited by high cost. Compared with airborne LiDAR, spaceborne LiDAR has the characteristics of large observation range and regular repeated observation. It has great advantages in quantitative inversion of forest parameters at large regional scale. It can not only reduce the consumption of manpower and time in field investigation, but also ensure accuracy, spatial integrity and time consistency (Yuan, 2022). At present, because the spaceborne LiDAR data can be used to detect the forest vertical structure in a large area, some scholars have applied it to the estimation of forest canopy height (Wang et al., 2023), forest height (Zhu, 2021; Lin et al., 2023; Luo et al., 2023), closure (Zhang et al., 2021) and biomass (Xu et al., 2023a; Song et al., 2022). ICESat-1 is the world’s first spaceborne LiDAR altimetry system. After its retirement in 2009, the United States launched ICESat-2 satellite in 2018. The photon counting laser altimeter on board adopts micro-pulse multi-beam photon counting LiDAR technology. These two satellites have been successfully applied to measure forest structure parameters, lake water level, glacier change and sea ice surface classification (Zhu, 2021; Song et al., 2022; Liu et al., 2023c; Petty et al., 2021; Cheng et al., 2023).

GEDI is the only full-waveform LiDAR system designed specifically to measure the vertical structure of vegetation that NASA launched at the end of 2018 (Xie et al. 2018). The difference between GEDI and other spaceborne LiDAR is its penetration ability in dense vegetation. The GEDI system consists of three lasers, one of which is divided into two beams (coverage beam), and the other two lasers maintain full power (full power beam). The coverage beam and the full power beam can penetrate 95% and 98% of the forest canopy to the ground, respectively (Liu et al., 2022). It is equipped with the world ‘s first multi-beam linear system laser altimeter for high-resolution forest vertical structure measurement, which is mainly used for accurate measurement of forest canopy height, vertical structure and ground elevation in tropical and temperate regions. From the GEDI waveform, four types of structural information such as landform, canopy height, canopy coverage and vertical structure can be extracted (Hang, 2022). GEDI is a full-waveform LiDAR data that can generate a forest canopy profile with a diameter of 25 meters. However, due to the fact that the GEDI footprints are distributed along the track and there is a certain distance between the footprints, the GEDI data is discrete and sparse. In order to overcome this limitation, previous studies have integrated continuous optical or SAR images with GEDI to infer GEDI samples spatially (Potapov et al., 2021). This extrapolation technique has been used in several studies to generate large-scale CHM and AGB maps. For example, Francini et al. (2022) integrated GEDI data and four decades of optical images to analyze forest disturbance and biomass changes in Italy, demonstrating that GEDI can capture changes in forest biomass caused by disturbance. Tamiminia et al. (2024) integrates the GEDI footprint with the Sentinel-2 image to generate a 10-meter wall to wall CHM of New York State in 2019, and then uses the generated CHM, Sentinel-1 and Sentinel-2 data to create a 10mAGB map of New York State in 2019. Guo et al. (2023) proposed a new method to integrate field measurement data, GEDI LiDAR, sentinel and terrain data to construct multi-source data-driven forest CHM and AGB models with a resolution of 30 m. Firstly, RFE-SVM method was used to determine the characteristics sensitive to forest height and AGB, and then three regression models were used to construct the CHM model. The GEDI point data was extended to the wall to wall CHM map, and finally the common features and measured data were selected to construct the model for estimating AGB. Although many scholars have done a lot of research on GEDI in forest attribute estimation, most of them are to evaluate forest canopy height or jointly estimate forest attributes with other remote sensing images. At present, no scholars have studied the effect of the number of GEDI light spots on the biomass estimation results.

The objective of this study is twofold. First, GEDI footprint of different orders of magnitude are evaluated and the optimal data set is selected. Secondly, the biomass was estimated by using the selected optimal footprint combined with the random forest model. Specifically, this paper aims to address the following research questions: 1) investigate the interpolation accuracy of GEDI footprint on different orders of magnitude. 2) explore the feasibility of using inverse distance weight interpolation to interpolate footprint spaceborne LiDAR samples. 3) and determine the optimal GEDI footprint as the input predictor of accurate AGB mapping.

## 2 Study area and research data

### 2.1 Study area

**Figure 1:**
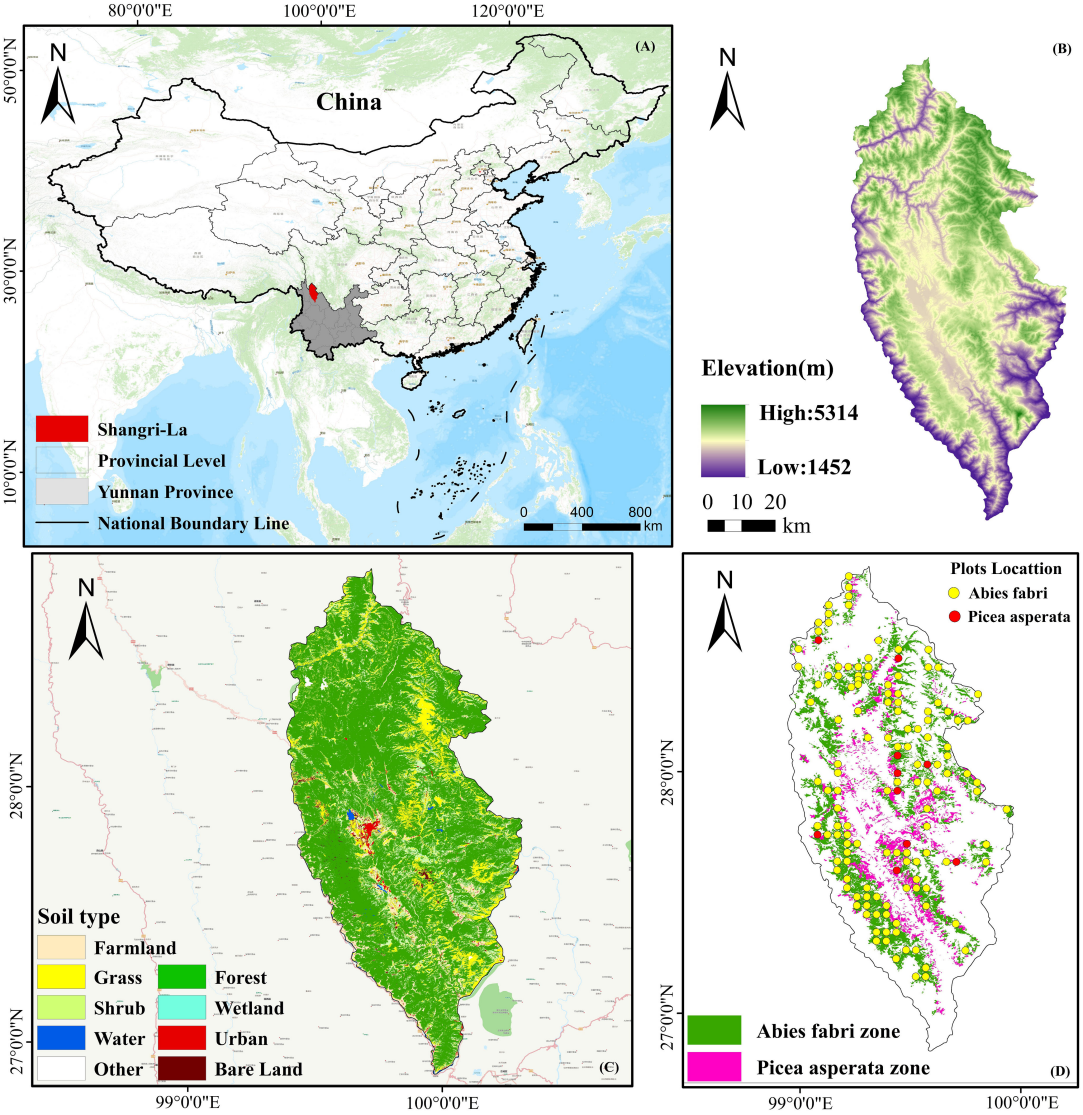
Geographical location of Shangri-La **(A)**; Altitude spatial variation **(B)**; Land use type **(C)**; Spruce-fir and sample site distribution **(D)**.

Shangri-La is located in the northwest of Yunnan Province and the eastern part of Diqing Tibetan Autonomous Prefecture, and characterized by a rugged internal terrain, mainly comprising a series of high mountains that stretch from the middle to the northeast (**Figure 1**). The altitude varies in the region, being higher in the northwest and lower in the southeast, with an overall altitude range of 1503 to 5545 meters, and an average altitude of approximately 3459 meters. The region experiences a mountainous cold temperate monsoon climate, with an average annual temperature of 5.4°C and an average annual precipitation of 618.4 mm. The frost-free period typically lasts from 129 to 197 days, with the rainy season occurring from July to September. This microclimate is attributable to its location in the transition zone between the subtropical evergreen broad-leaved forest vegetation area of Yunnan and the alpine vegetation area of the Qinghai-Tibet Plateau. As a result, the vegetation distribution varies significantly between the north and the south, with distinct vertical structure characteristics prevailing in the region. The distribution of the species varies based on altitude. At 1500 ~ 2800 m, its primary habitat is found within warm-temperate coniferous forests, where *Pinus armandii* and *Pinus yunnanensis* dominate. As the altitude ranges from 2800~3500 m, the species tends to thrive in warm and cool coniferous forests, with *Tsuga yunnanensis* and *Pinus densata* as the dominant species. Moving further up, at an altitude of 3200 ~ 4200 m, the species is predominantly distributed in cold-temperate coniferous forests, where *larix potaninii* var.*macrocarpa*, *picea brachytyla* var.*complanata*, and *longbract fir* are the dominant species. Above 4200 m, the distribution extends to cold-temperate shrubs, particularly *rhododendron delavayi*, and *meadows*.

### 2.2 Field inventory data and calculation of AGB

Forest resources survey data used in our study. For the purpose of this study, we focused on the two dominant tree species, Picea asperata and Abies fabri, totaling 138 plots, in order to compute the biomass. To determine the total biomass of each sample, it is necessary to calculate the biomass of individual trees. Consequently, the cumulative biomass of all individual trees within the sample represents the total forest biomass of the sample. Common standing tree biomass models mainly include one-dimensional, binary and multivariate biomass models. The one-dimensional biomass model mostly selects the DBH of the tree as an independent variable, and the binary biomass model selects the DBH and tree height (Wang et al., 2018). In this study, the biomass in the plot was calculated by referring to the binary biomass model provided by the forestry industry standards such as ‘Tree biomass models and related parameters to carbon accounting for Picea’ (Zeng et al., 2016a), ‘Tree biomass models and related parameters to carbon accounting for Abies’ issued by the State Forestry Administration (Zeng et al., 2016b). Based on the data of tree height and DBH of forest resource survey data, the aboveground biomass values of spruce-fir in the plot were calculated by equations, respectively. The AGB of the sample plot is divided by the area, which can be converted into the AGB per hectare, and the unit biomass information of the sample plot is obtained as shown in **Table 1**.

**Table 1:** Spruce-fir plot information summary.

Number of plots	Maximum (t/hm^2^)	Minimum (t/hm^2^)	Average (t/hm^2^)	Median (t/hm^2^)	Standard error (t/hm^2^)
139	296.09	5.19	101.42	92.73	65.09


$${\text{M}}_{\text{spruce}}=0.09152{\text{D}}^{2.21060}{\text{H}}^{0.25663}$$

$${\text{M}}_{\text{fir}}=0.06127{\text{D}}^{2.05753}{\text{H}}^{0.50839}$$

Where ${\text{M}}_{\text{spruce}}$ the model of aboveground single is tree biomass of *spruce*, and ${\text{M}}_{\text{fir}}$ is the model of the aboveground single tree biomass of *fir*. M is the AGB; D is the diameter at breast height; H is the single tree height.

### 2.3 GEDI data

GEDI spaceborne LiDAR is a form of active remote sensing technology that captures extensive data by emitting short-wavelength laser pulses to penetrate the forest canopy and retrieve precise three-dimensional forest structure information (Saarela et al., 2022). Comprising three lasers, two of which are full-power lasers and the remaining one functioning as a covering laser, GEDI utilizes beam jitter to split into two beams, resulting in a total of eight beams. The footprint diameter is approximately 25 m, with a footprint spacing of 60 m along the track and 600 m spacing between adjacent ground tracks. The overall width of the laser trajectory is 4200 m (Potapov et al., 2021). GEDI data is composed of discrete footprints, each containing various echo indicators for feature extraction and input into forest biomass models. These echo indicators are crucial for subsequent GEDI data analysis and interpretation. In this study, the data product of the spaceborne LiDAR GEDI Level 2B Version 2 is utilized. GEDI Version 2 enhances geo-positioning accuracy, updates metadata to include spatial coordinates, and enables querying in NASA Earth data retrieval. The average geolocation error of version 2 data product is 10.3 m, showing a 50% improvement in geolocation accuracy compared to Version 1. GEDI contains four levels of products: Level 1 encompasses geolocated return energy waveform, Level 2 includes footprint level canopy cover and vertical profile metrics, Level 3 comprises gridded land surface metrics, and Level 4 consists of footprint level and gridded aboveground biomass density (Tian, 2023). All GEDI data used in this study are sourced from Earthdata (https://www.earthdata.nasa.gov), with the data acquisition time ranging from April 23, 2019 to December 04, 2019.

## 3 Methods

**Figure 2:**
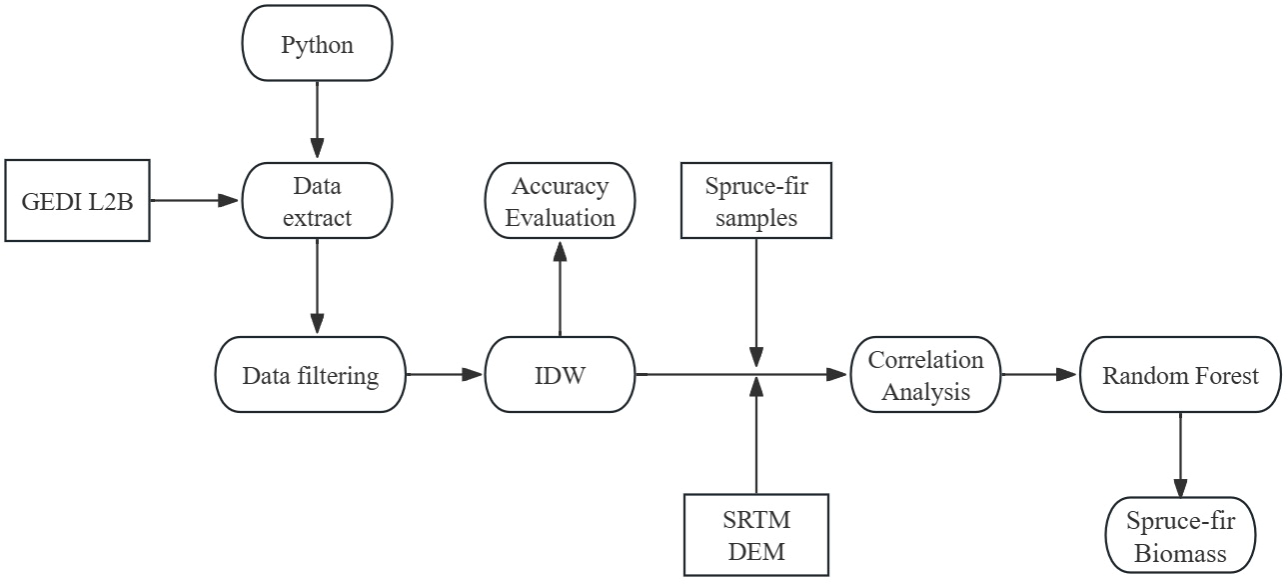
Steps used to estimate AGB using GEDI data.

The implementation of this method encompasses five steps, as described in the flowchart of **Figure 2**: 1) GEDI Data Filtering; 2) GEDI Footprint Distribution and Interpolation Results Analysis; 3) Accuracy Evaluation of GEDI Footprint Interpolation Results; 4) Correlation Analysis between GEDI Variables and Biomass; 5) Biomass Modeling.

### 3.1 Inverse distance weighting model

The inverse distance weighting method (IDW) is based on the assumption that nearby things are more similar to each other than those that are farther apart. It considers that each measurement point has a local influence, and that this influence gradually diminishes as the distance between point’s increases. In essence, IDW assigns a weight to each interpolation point by taking the reciprocal of the distance between the interpolation point and the measured point to perform a weighted average. This method is particularly suitable for situations where measurement sites are evenly distributed and of high density.


$${\text{Z}}^{*\;}\left({\text{x}}_{0}\right)=\sum_{\text{i}=1}^{\text{N}}{\text{λ}}_{\text{i}}\text{Z}\left({\text{x}}_{\text{i}}\right)=\sum_{\text{i}=1}^{\text{N}}\left(\frac{{\text{d}}_{\text{i}0}^{-\text{p}}}{\sum_{\text{J}=1}^{\text{N}}{\text{d}}_{\text{i}0}^{-\text{p}}}\right)\text{Z}\left({\text{x}}_{\text{i}}\right)$$

where $Z^{*\;}\left(x_{0}\right)\;$ is the interpolation result of the interpolation point $x_{0}$ ; $Z\left(x_{i}\right)$ is the measured value of the measured point $x_{i}$ ; n is the number of measured points involved in the calculation; ${\text{λ}}_{\text{i}}$ is the weight coefficient;. is the distance between the measured point $x_{i}$ and the interpolation point $x_{0}$ ; p is the power of distance, generally p =1 or p =2.

### 3.2 Evaluation of interpolation accuracy

The accuracy of **interpolation** was assessed by comparing the root mean square error (RMSE) and the coefficient of determination (R^2^) between the measured and predicted values at both the interpolated footprints and the verification sample points. R^2^ quantifies the correlation between the predicted and estimated values, with a value closer to 1 indicating higher model accuracy. On the other hand, RMSE primarily elucidates the estimation’s sensitivity to extreme effects within the sample data. The mean absolute error (MAE) calculates the average difference between predicted and measured values. A smaller MAE indicates a more accurate prediction model.


$${\text{R}}^{2}=1-\frac{\sum_{\text{i}=1}^{\text{n}}{\left({\hat{\text{y}}}_{\text{i}}-{\text{y}}_{\text{i}}\right)}^{2}}{\sum_{\text{i}=1}^{\text{n}}{\left({\hat{\text{y}}}_{\text{i}}-{\bar{\text{y}}}_{\text{i}}\right)}^{2}}$$

$$\text{RMSE}=\sqrt{\frac{\sum_{\text{i}=1}^{\text{n}}{\left({\hat{\text{y}}}_{\text{i}}-{\text{y}}_{\text{i}}\right)}^{2}}{\text{n}}}$$

$$\text{MAE}=\frac{1}{n}\sum_{i=1}^{n}\left|{\hat{y}}_{i}-y_{i}\right|$$

where n is the number of footprints, ${\hat{\text{y}}}_{\text{i}}$ is the predicted value on the footprint i, ${\text{ y}}_{\text{i}}$ is the observed value on the footprints, and ${\bar{\text{y}}}_{\text{i}}$ is the arithmetic mean of the observed value.

### 3.3 Biomass estimation model

#### 3.3.1 Support vector machine

Support Vector Machine (SVM) is an efficient machine learning algorithm, which is based on the principle of dividing hyperplanes. The core idea of this classifier is to divide the data into several possible categories by constructing a hyperplane. By continuously adjusting and optimizing this hyperplane, the overall deviation is minimized (Chen et al., 2023). For the known training data set, the SVM algorithm will find the hyperplane that maximizes the classification interval, and use these intervals to predict the new data set. A hyperplane is a character space one dimension less than its surrounding planes. In the two-dimensional data set, the straight line is a hyperplane. The key to its core idea is to map complex nonlinear data onto a high-dimensional array, a mapping process called kernel learning (Qiu et al., 2020). In this way, the data can be regarded as a linear function in the high-dimensional feature space, while the nonlinear features are mapped to the low-dimensional space. In this study, the e1071 package in R language was used, the kernel function was radial basis, the C of spruce-fir was 1, and the g was 0.92.

#### 3.3.2 K-nearest neighbor algorithm

K nearest neighbor regression (KNN) is a typical nonparametric algorithm. It is a univariate or multivariate prediction method based on the spatial similarity between observation points and prediction points. It can be used for classification and parameter estimation problems, especially for remote sensing data with non-normal or unknown probability density distribution function (Zhang et al., 2022). As a non-parametric learning method widely used in scientific research and engineering technology, its core is to use the observation point data in the nearest k neighborhoods to predict the value of unknown points. This algorithm can effectively deal with remote sensing data that are affected by the non-normal distribution of the probability density distribution function (PDF) or unknown distribution, and realize the classification task and parameter estimation of the data by analyzing the correlation between the observed value and the predicted point (Wu et al., 2024). In this paper, the knn function in the class package of R language is used to implement the algorithm, and the K value of spruce and fir is set to 9 respectively.

#### 3.3.3 Random forest regression

Random forest is a machine learning algorithm that exhibits high accuracy and robustness, making it suitable for analyzing high-dimensional and highly correlated data sets (Wei et al., 2024). The algorithm operates on the principle of utilizing the bootstrap method to iteratively and randomly sample from the original data, constructing decision trees for each sample. Through the combination of predictions from multiple decision trees, the final prediction results are determined by collective voting (Lourenço et al., 2021). This approach eliminates the necessity for feature selection and enables the handling of high-dimensional data. Moreover, the random sampling of instances leads to the utilization of different training sets for each decision tree, thereby mitigating overfitting to a certain extent (Yang et al., 2021a). Notably, the Random Forest package within R software facilitates predictive regression using the random forest algorithm, necessitating adjustments to the number of decision trees (ntree) and the number of variables extracted during tree splitting (mtry). The ntree is set to 300 and the mtry is set to 2.

### 3.4 Biomass model validation

To improve the accuracy of the biomass inversion model, it is essential to select the optimal variables that are conducive to biomass inversion while avoiding data redundancy, which can diminish the accuracy of model inversion. This process not only reduces the running time of the algorithm, but also prevents over-fitting caused by model complexity. Through an analysis of the correlation between independent variables and the biomass of spruce-fir, the first five variables with the highest correlation were identified and selected as the determinants of the model. These variables were utilized to estimate the biomass of spruce-fir within the study area, and their accuracy was subsequently evaluated through cross-validation (Vaithiyanathan and Sudalaimuthu, 2022). This method ensures that the model is based on the most relevant data, leading to an improved accuracy in biomass estimation.

To ensure the stability of the model and reduce accidental error caused by the division of training and verification samples, we employed the ten-fold cross-validation method in this study. The data set, containing 138 ground observation samples, was divided into 10 groups with an equal number of samples. Each group was subsequently utilized as the verification set in turn to assess the model’s estimation ability, while the remaining 9 groups served as the training set for constructing the model. For each cross-validation, we calculated the determination coefficient (R^2^), the root mean square error (RMSE) and mean absolute error (MAE) between the predicted and measured values. This process was repeated 10 times, and the accuracy of each model was evaluated based on the average R^2^, RMSE and MAE values. The calculation formulas for R^2^, RMSE and MAE can be found in 3.2 sections.

## 4 Results

### 4.1 GEDI preprocessing and data filtering

#### 4.1.1 GEDI data pre-processing

GEDI is a geolocation waveform product, which contains 8 beams and 1 metadata. Each beam group stores the transmitted and received waveforms, geolocation parameters and geophysical correction parameters obtained by the beam after geolocation correction (Dubayah et al., 2021). GEDI uses 1064nm **laser** pulse to measure the vertical structure of the forest, and the obtained waveform needs a series of processing to obtain the vertical structure parameters of the forest. According to the theoretical basis file of GEDI official algorithm, a Gaussian filter (smoothing width) with a width of 6.5ns is first used to smooth the waveform to reduce the noise in the signal without affecting the signal in the waveform (Schwartz et al., 2024). After smoothing, the two positions represented as searchstart and searchend in the waveform are determined. Searchstart and searchend are the first and last positions of the signal with a signal strength higher than the following thresholds:


threshold=mean+c*std

Where ‘threshold’ is the background threshold, ‘mean’ is the average noise level, ‘std’ is the standard deviation of the smoothed waveform noise, and ‘c’ is a given constant, which is stored in the data-aided group of waveform processing as back_threshold or front_threshold.

**Figure 3:**
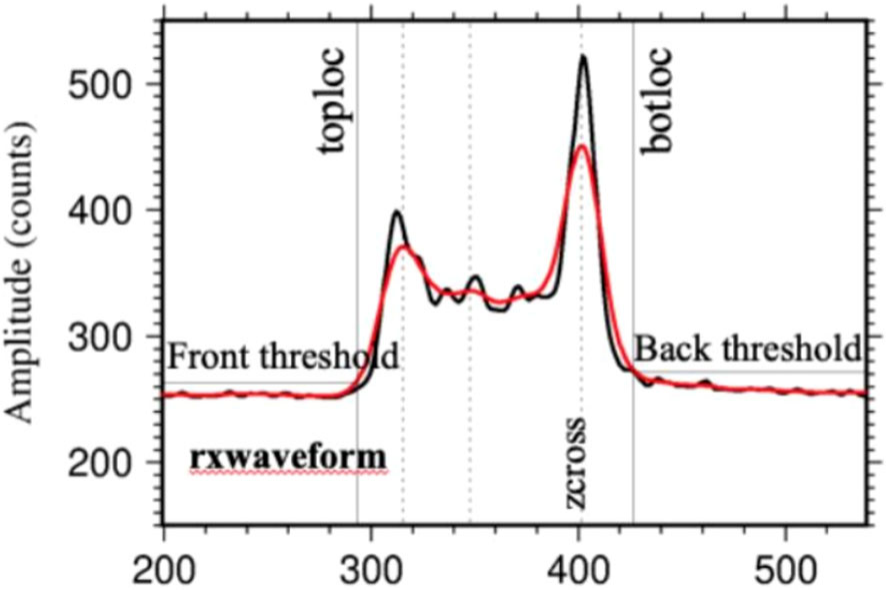
Received waveform before and after GEDI fitting (Dubayah et al., 2020).

After determining the positions of searchstart and searchend, the region between them (i.e., waveform range) is extended to the expected length, and the highest (toploc) and lowest (botloc) detectable echoes are determined within the waveform range. toploc and botloc represent the highest and lowest positions in the waveform range when the two adjacent intensities are higher than the threshold, respectively. At this time, the threshold equations used to determine toploc and botloc are the same as the above equations. As shown in **Figure 3**, the black waveform is the waveform before Gaussian fitting, and the red waveform is the waveform after Gaussian fitting. After fitting, the burr part of the waveform is removed, and a smoother waveform is obtained, while reducing the influence of noise on the waveform. Subsequently, the second filtering is performed on the waveform part whose intensity exceeds the threshold to determine the position of different peaks in the waveform, such as the ground peak or the peak at the top of the crown. The width of the second Gaussian filter is expressed as smoothwidth cross. Finally, the position of the final detected peak is used to determine the position of the ground echo in the waveform. After smoothing and filtering the waveform data stored in the L1B product, the results such as noise mean, noise standard deviation, waveform processing-related parameter settings, and geolocation parameters are output and stored in the L2B product.

#### 4.1.2 GEDI data filtering

In the range of Shangri-La, python software is used to write code to extract and filter the footprints according to the position and parameter name of each parameter in the GEDI L2 B data source file, and convert it into a Shapefile format that can be processed by ArcGIS. In this study, 14 modeling parameters were extracted from GEDI_L2B data products, which are cover, dem, fhd_normal, Landsat_treecover, leaf_off_doy, leaf_on_doy, modis_treecover, modis_nonvegetated, pai, pgap_thea, rg, rh100, rv and sensitivity. The specific names of these 14 parameters and their corresponding descriptions are listed in **Table 2**. Due to the lack of information or poor quality of some footprints, not all waveform data can meet the forest resource survey. Therefore, when processing data, three values (sensitivity, degrade_flag, quality_flag) of GEDI’ s own quality characteristics are used to screen out the spots that meet the conditions. A quality_flag value of 1 indicates the laser shot meets criteria based on energy, sensitivity, amplitude, real-time surface tracking quality and difference to a DEM. Sensitivity is defined as the difference between the ratio of the ground echo area to the total echo area and 1, and its value range is 0 ~ 1. Different surface coverage types have different values, and the general forest is set to 0.9. Degrad_flag indicates the degradation flag of the satellite pointing to the positioning state, and the footprint with degrad_flag of 0 needs to be retained (Jiang et al., 2021).

**Table 2:** GEDI parameters information.

Parameters name	description	Parameters name	description
cover	Total canopy cover,	DEM	Digital elevation model
fhd_normal	Foliage Height Diversity index calculated by vertical foliage profifile normalized by total plant area index	landsat_treecover	Landsat tree canopy cover
leaf_off_doy	Leaf off day of year	leaf_on_doy	Leaf on day of year
modis_treecover	Percent tree cover from MODIS data	modis_nonvegetated	Percent non-vegetated from MODIS data
pai	Total plant area index	pgap_thea	Estimated Pgap(theta) for the selected L2A algorithm.
rg	Integral of the ground component in the RX waveform	rh100	Height above ground of the received waveform signal start
rv	Integral of the vegetation component in the RX waveform	sensitivity	Maximum canopy cover that can be penetrated considering the SNR of the waveform
degrad_flag	Non-zero values indicate the shot occurred during a degraded period. A non-zero tens digit indicates degraded attitude, a non-zero ones digit indicates a degraded trajectory.	quality_flag	Flag simplifying selection of most useful data for Level 2B

### 4.2 GEDI data distribution analysis

To verify the effect of footprints interpolation of different data and achieve a random and uniform distribution of footprints, our study utilized a Python algorithm to extract representative footprints at intervals of 10, 30, 50, 70, and 100 shots when extracting GEDI data. The extracted data is presented in **Table 3**, which reveals that 13,127 footprints were extracted at intervals of 10 shots, and 4,343 footprints were extracted at intervals of 30 shots. Similarly, 2,646 footprints were obtained at intervals of 50 shots, while 1,871 and 1,309 footprints were obtained at intervals of 70 and 100 shots, respectively.

**Table 3:** The Number of Spots with different shot intervals.

Shot interval	10	30	50	70	100
Number of footprints	13127	4343	2646	1871	1309

**Figure 4:**
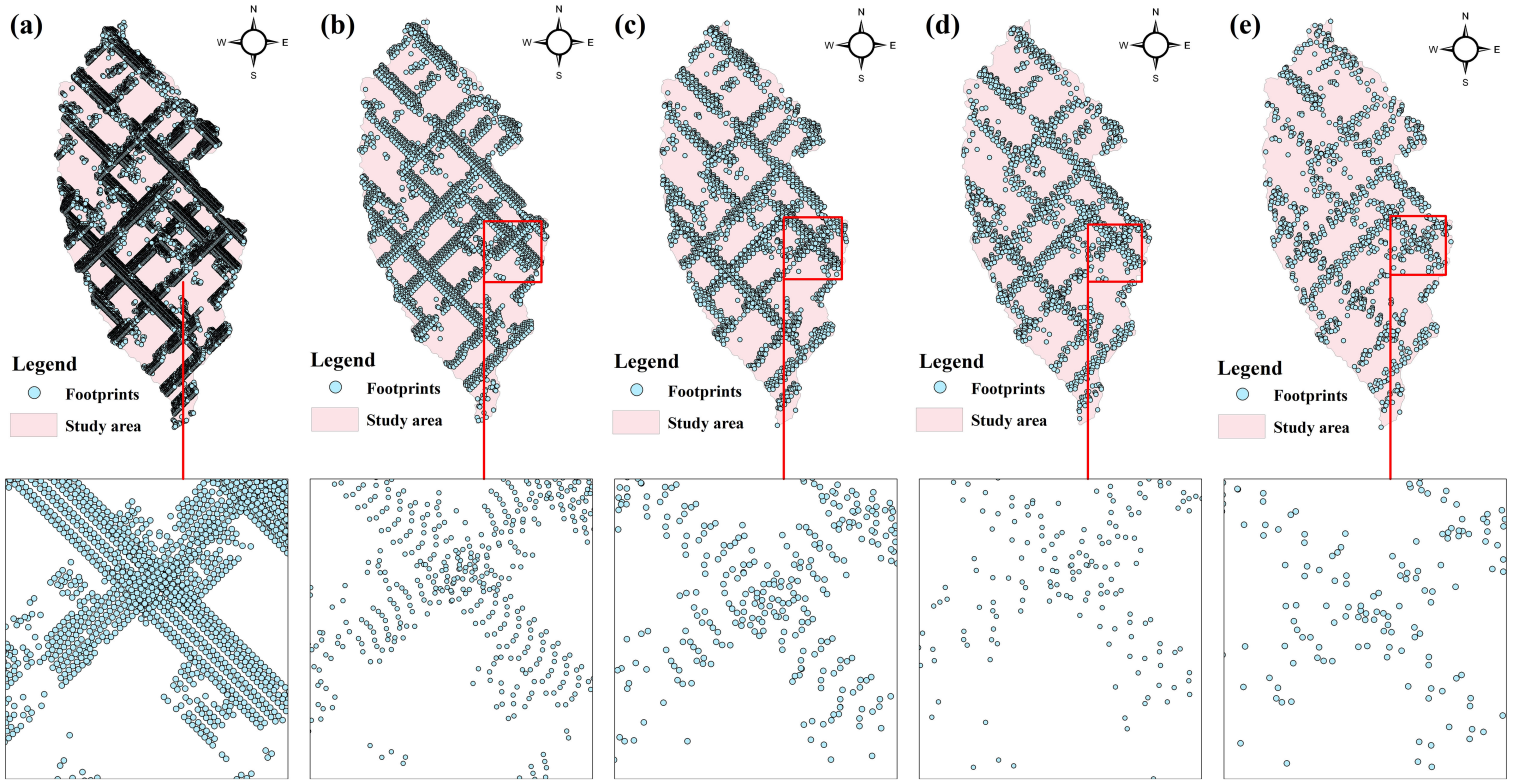
The distribution of different number of footprint points, **(A)** is 10 shots, **(B)** is 30 shots, **(C)** is 50 shots, **(D)** is 70 shots and **(E)** is 100shots.

The footprints distribution map can be obtained by visualizing the extracted footprints. **Figure 4** illustrates the distribution of footprints extracted at intervals of every 10, 30, 50, 70, and 100 shots (as denoted by a-e in **Figure 4**). The characteristics of GEDI’s laser scanning are evident in the distribution of GEDI footprints, demonstrating the formation of footprints lattices as a result of orbit overlap. Dense footprints are primarily concentrated around the lattice, with no footprints inside. It is apparent from **Figure 4** that an increase in the number of footprints results in closer distances between footprints, leading to a more pronounced lattice effect, especially in **Figure 4A**. Conversely, a decrease in the number of footprints significantly reduces the density of footprints on the same track, as depicted in **Figure 4E**, subsequently weakening the lattice effect and evoking a more random distribution pattern.

### 4.3 GEDI footprint interpolation results

**Figure 5:**
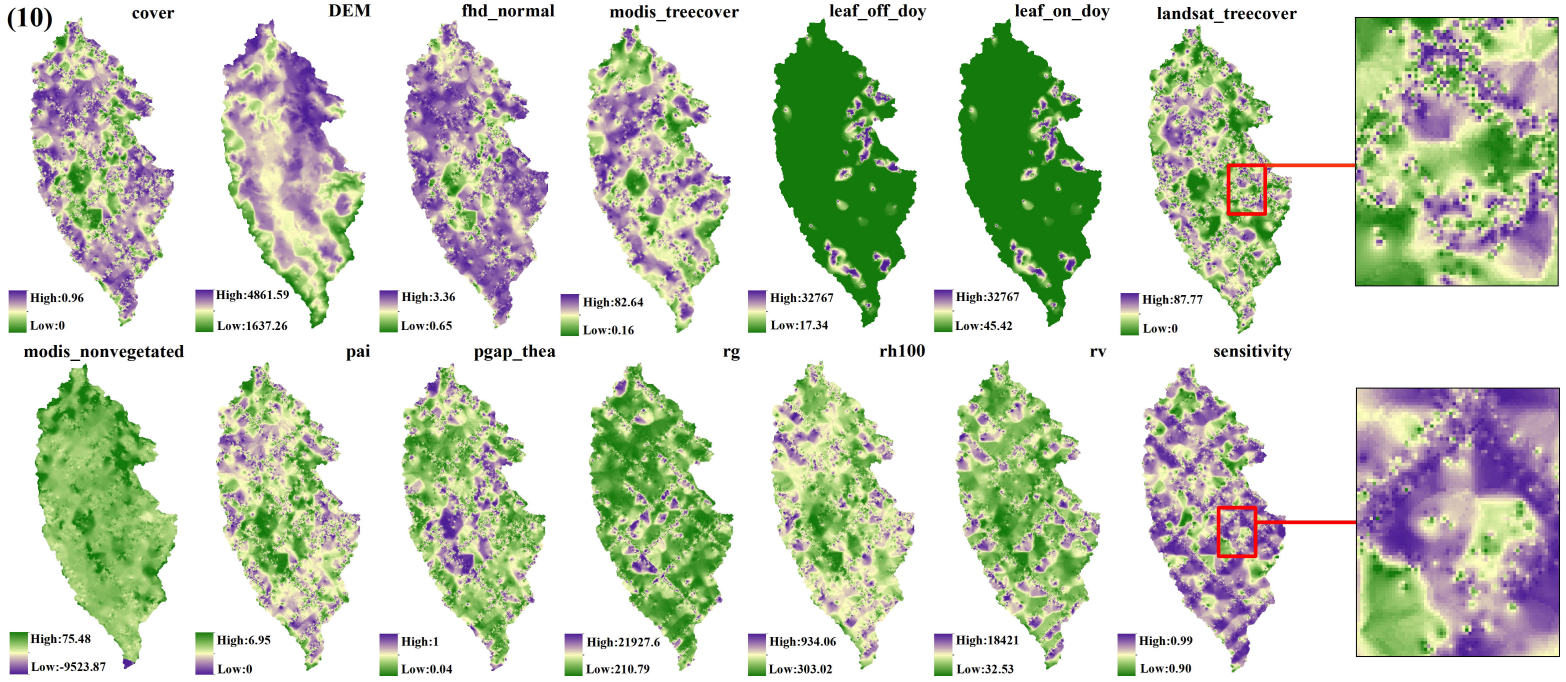
GEDI parameter interpolation results with a shot interval of 10.

**Figure 6:**
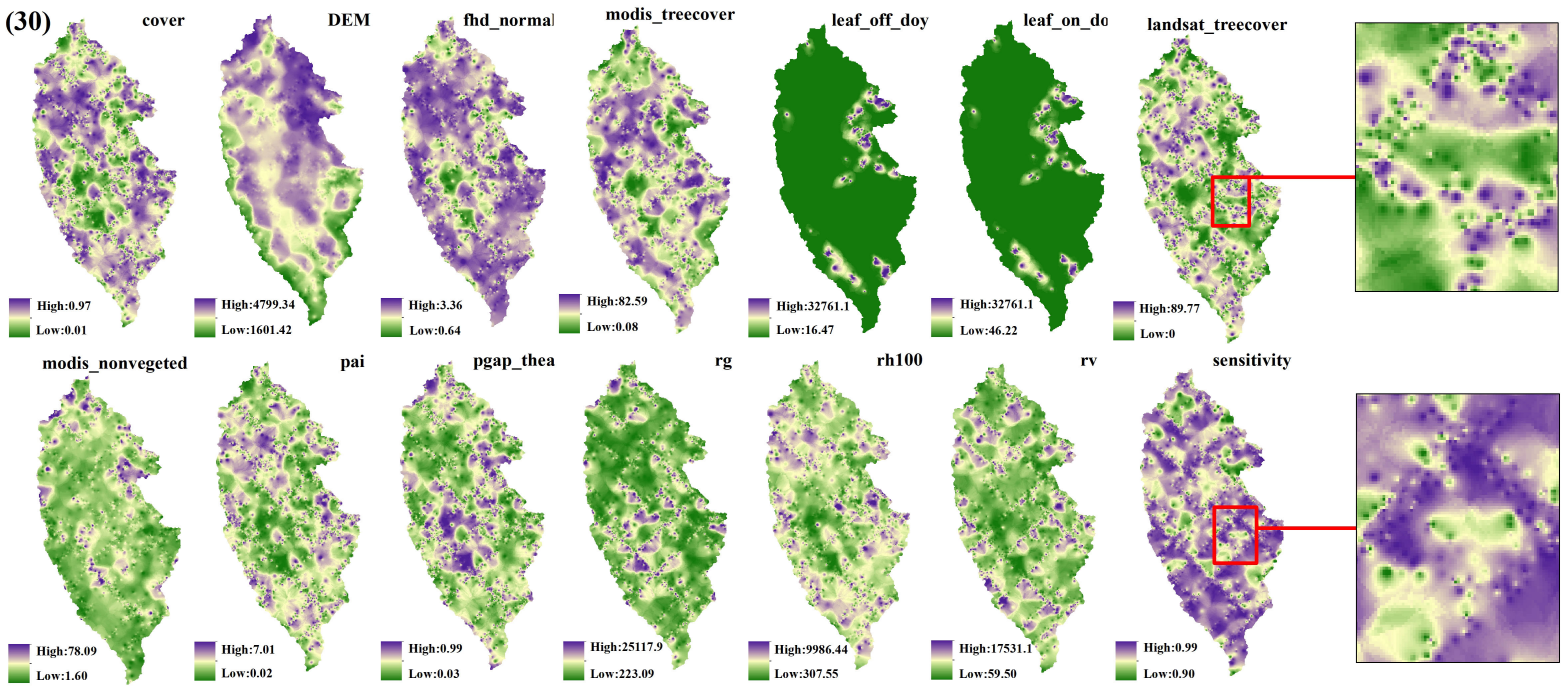
GEDI parameter interpolation results with a shot interval of 30.

**Figure 7:**
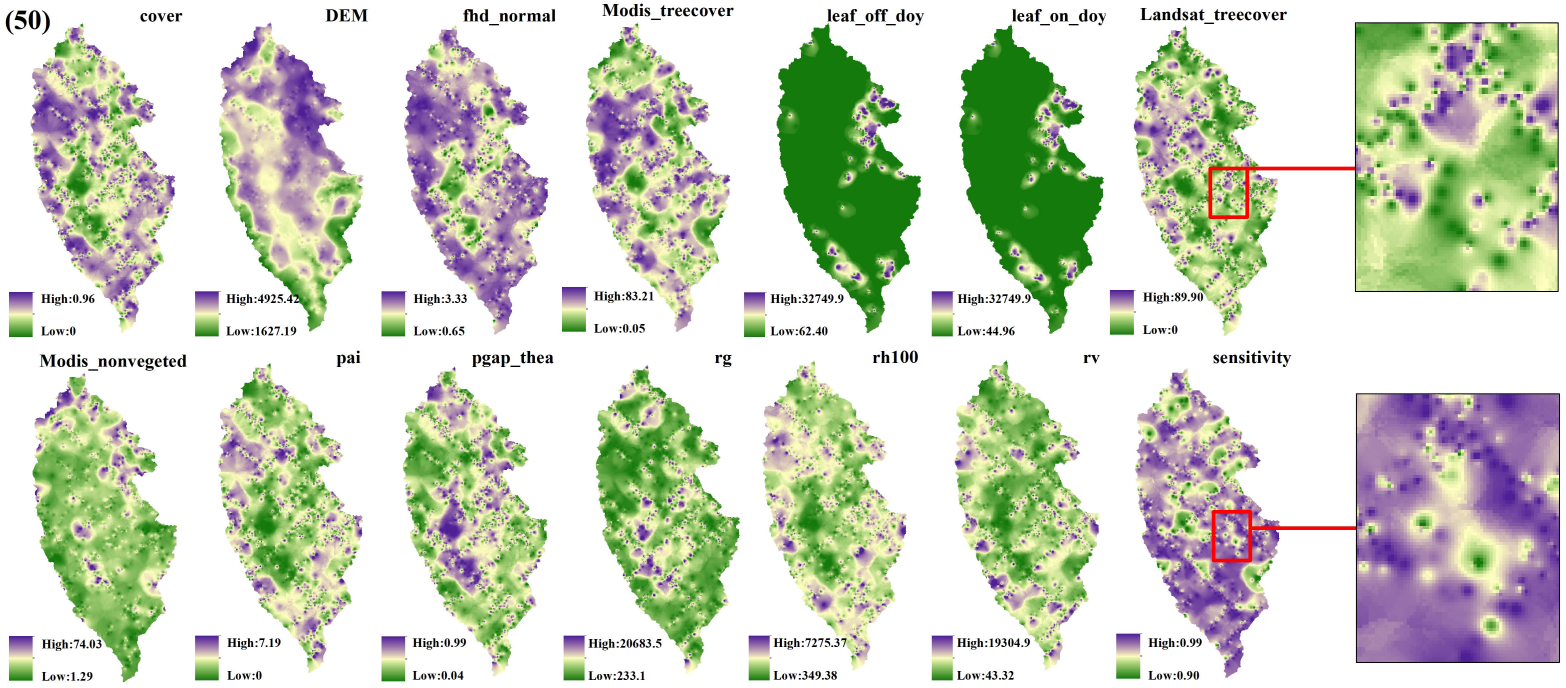
GEDI parameter interpolation results with a shot interval of 50.

**Figure 8:**
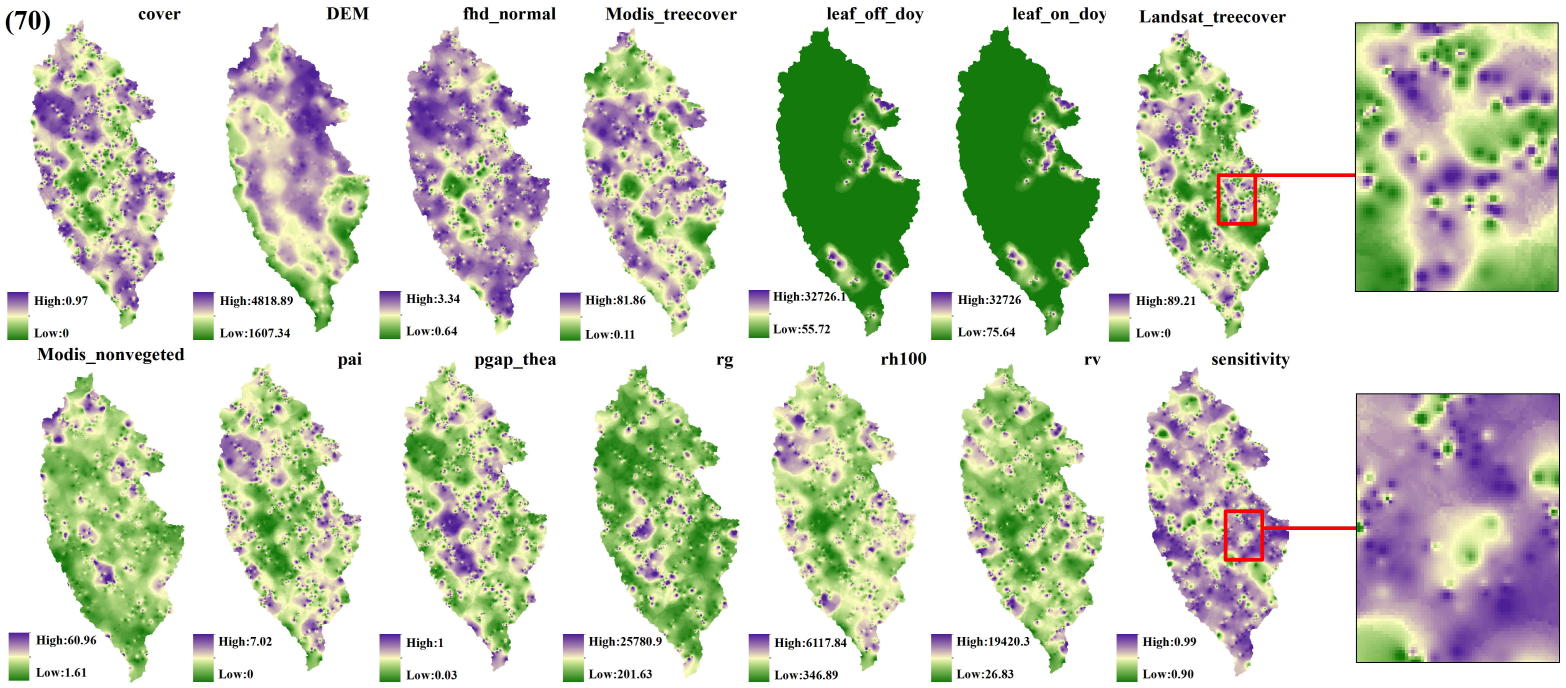
GEDI parameter interpolation results with a shot interval of 70.

**Figure 9:**
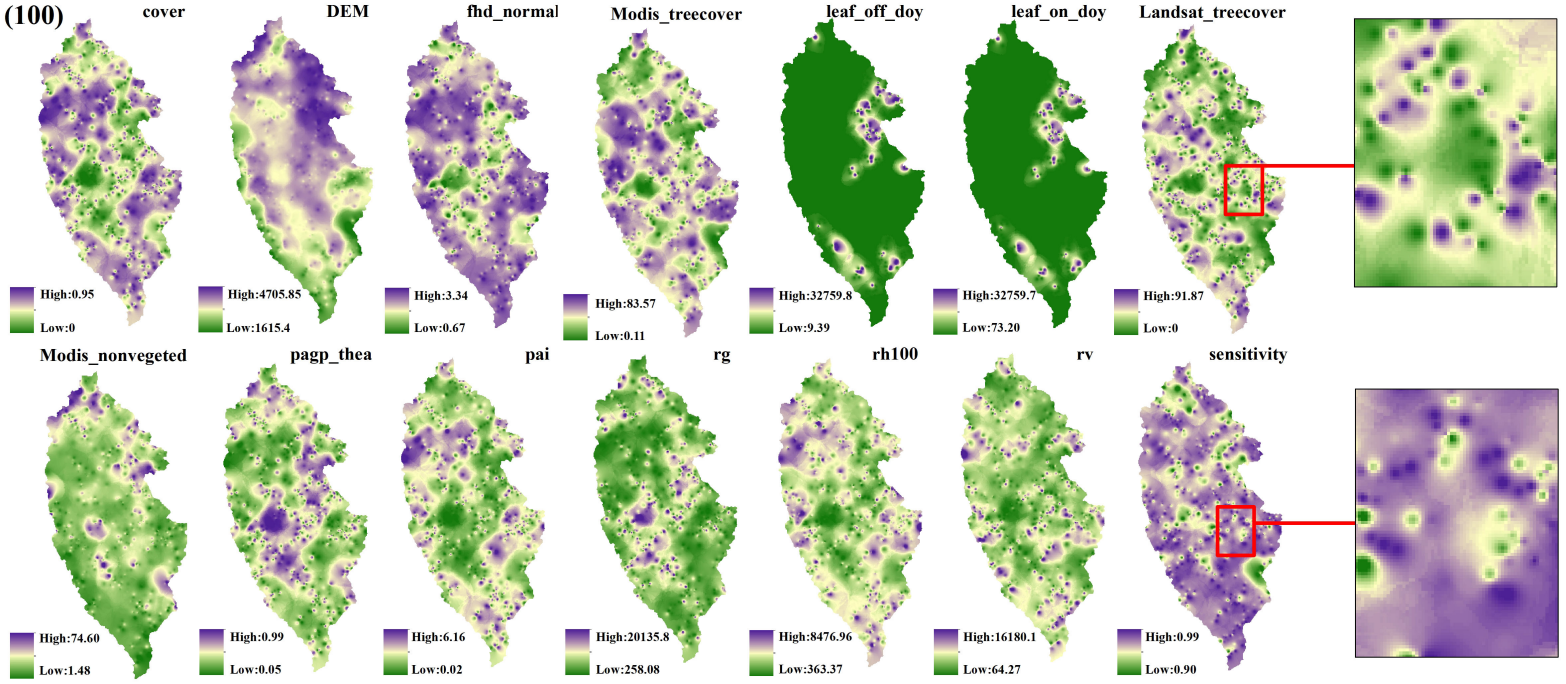
GEDI parameter interpolation results with a shot interval of 100.

The spatial interpolation prediction of independent variables extracted from different numbers of GEDI footprints is depicted in **Figures 5**–**9**. Overall, the spatial distribution of the independent variable prediction does not exhibit significant differences across the five orders of magnitude of footprints. However, variations are evident in the specific areas where the footprints are located. The prediction map (**Figures 5**–**7**) derived from the interpolation of the independent variables with shot intervals of 10, 30, and 50, demonstrates a lack of smoothness, characterized by the presence of a “bull’s-eye” phenomenon, which allows for clear visibility of the footprint positions (For example, the local magnification of sensitivity and Landsat_treecover in **Figures 5**–**7**). Moreover, the distribution of GEDI orbit reveals discernible staggered bands in the prediction results of four specific independent variables – cover, Landsat_treecover, pgap_thea, and pai. This observation may be attributed to the dense distribution and substantial number of footprints along the track. In contrast, the four independent variables of dem, modis_nonvegeted, leaf_on_doy, and leaf_off_doy yield relatively smooth prediction results with minimal error, regardless of the magnitude of the prediction, compared to other variables.

The estimation results of different independent variables have changed as the number of predicted footprints decreases. **Figures 8**, **9** demonstrates that the previously observed staggered bands in the prediction results of each variable have notably reduced, albeit the persisting presence of point-like ‘salt and pepper’ high-value points in the figure (Such as the local magnification of sensitivity and Landsat_treecover in **Figures 8**, **9**). A comparison of the spatial prediction maps of the five quantities of independent variables reveals smoother interpolation results for the footprints extracted every 100 shots, with a relatively continuous spatial distribution map. These results exhibit more gradients and smoother edges compared to the other five respective results. Notably, among the interpolation results of different numbers of footprints, those extracted every 100 shots yield the best outcomes, followed by 70 and 50, with 30 and 10 producing the least favorable results.

### 4.4 Accuracy evaluation of GEDI footprint interpolation results

To evaluate the accuracy of the GEDI footprint extraction, the data was divided into two parts: 70% for interpolation and 30% for accuracy evaluation. The study area’s accuracy of spatial prediction for independent variables at different sampling densities is measured through the R^2^, RMSE and MAE of the verification set (consisting of 3938, 1303, 794, 561, and 275 footprints). It can be seen from **Table 4** that the R^2^ of the independent variable increases with the decrease of the sampling density, while the values of RMSE and MAE generally decrease with the decrease of the sampling density, indicating that the prediction accuracy increases with the decrease of the number of light spots. This trend suggests that prediction accuracy improves with decreasing footprints. Notably, the group with the highest R^2^ and lowest RMSE and MAE is the one with extractions made every 100 shots, followed by 70 and 50 shots. The prediction effect for footprints extracted every 10 shots is the poorest, with the lowest R^2^ observed for pai, Landsat_treecover, pgap_thea, and cover recorded at 0.47, 0.47, 0.49, and 0.51, respectively. These accuracy results align with the interpolation findings in **Figure 4** to **Figure 8**, demonstrating that lower accuracy in variable interpolation leads to a more pronounced band phenomenon along the footprint distribution.

**Table 4:** Different GEDI footprints interpolation R^2^, RMSE and MAE.

parameter name	Different GEDI footprints interpolation R^2^, RMSE and MAE														
	10			30			50			70			100		
	R^2^	RMSE	MAE	R^2^	RMSE	MAE	R^2^	RMSE	MAE	R^2^	RMSE	MAE	R^2^	RMSE	MAE
pai	0.47	1.23	0.90	0.61	1.04	0.70	0.62	1.03	0.67	0.63	1.02	0.64	0.94	0.44	0.26
Modis_treecover	0.88	8.90	5.60	0.79	11.56	7.76	0.7	13.14	7.68	0.77	12.10	7.98	0.96	5.34	3.10
Modis_nonvegeted	0.71	6.15	4.13	0.73	5.92	3.55	0.75	5.81	3.31	0.69	6.17	3.34	0.97	2.2	1.98
Landsat_treecover	0.47	25.09	18.45	0.6	21.92	14.08	0.6	21.35	14.66	0.66	20.42	13.43	0.93	9.44	5.39
Leaf_off_doy	0.8	3582.7	889.6	0.78	3144.4	686.25	0.8	3086.9	711.47	0.87	2618.5	715.29	0.98	1420.5	349.48
Leaf_on_doy	0.71	3590.4	893.9	0.78	3151.8	690.23	0.8	3093.0	715.45	0.87	2625.3	718.96	0.98	1425.4	351.19
Fhd_normal	0.56	0.39	0.26	0.71	0.32	0.20	0.68	0.34	0.20	0.67	0.34	0.19	0.96	0.12	0.07
DEM	0.97	108.6	76.23	0.96	118.95	73.42	0.95	129.96	77.44	0.94	146.82	83.13	0.99	55.33	35.49
cover	0.51	0.22	0.17	0.63	0.19	0.13	0.61	0.21	0.13	0.64	0.19	0.12	0.94	0.08	0.05
rg	0.52	3041.9	2010.7	0.67	2543.7	1585.2	0.6	2565.3	1564.4	0.68	2610.0	1491.8	0.95	1032.1	563.69
Pgap_thea	0.49	0.22	0.17	0.65	0.19	0.13	0.65	0.19	0.13	0.69	0.12	0.12	0.9	0.08	0.05
Sensitivity	0.57	0.014	0.01	0.61	0.013	0.01	0.65	0.012	0.01	0.68	0.011	0.01	0.92	0.004	0.00
rv	0.54	2799.5	1899.3	0.67	2340.7	1492.3	0.68	2356.7	1478.7	0.62	2361.2	1388.2	0.89	1007.8	583.94
Rh100	0.55	721.1	505.81	0.63	638.88	389.63	0.62	648.05	391.44	0.67	612.99	382.29	0.96	224.04	139.90

### 4.5 Correlation analysis between GEDI variables and biomass

**Figure 10:**
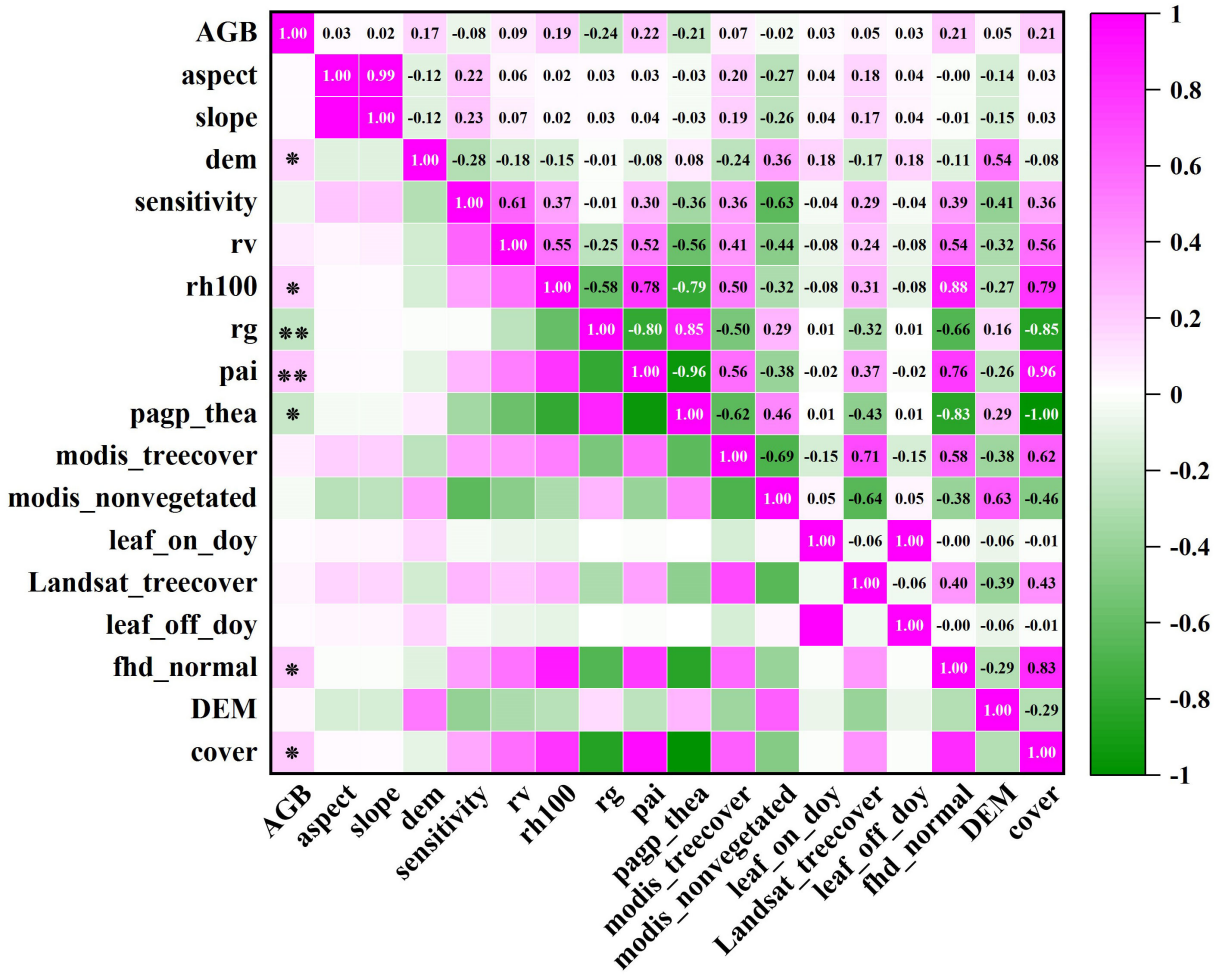
GEDI parameters and spruce-fir AGB correlation coefficient matrix. ‘ ** ‘ was significantly correlated at the 0.01 level (bilateral), and ‘ * ‘ was significantly correlated at the 0.05 level (bilateral).

In section 3.2, the footprint extracted from every 100th shot is found to have the best interpolation effect of GEDI independent variables. Consequently, the 916 footprint independent variables are chosen for biomass estimation. In conjunction with Shangri-La’s topographic factors, a correlation analysis is performed with the biomass of Shangri-La spruce-fir, and the variables showing the highest correlation are then identified as the modeling indicators. The correlation coefficient matrix presented in **Figure 10** displays the Pearson correlation coefficients between all remote sensing variables and forest aboveground biomass (AGB).

**Figure 11:**
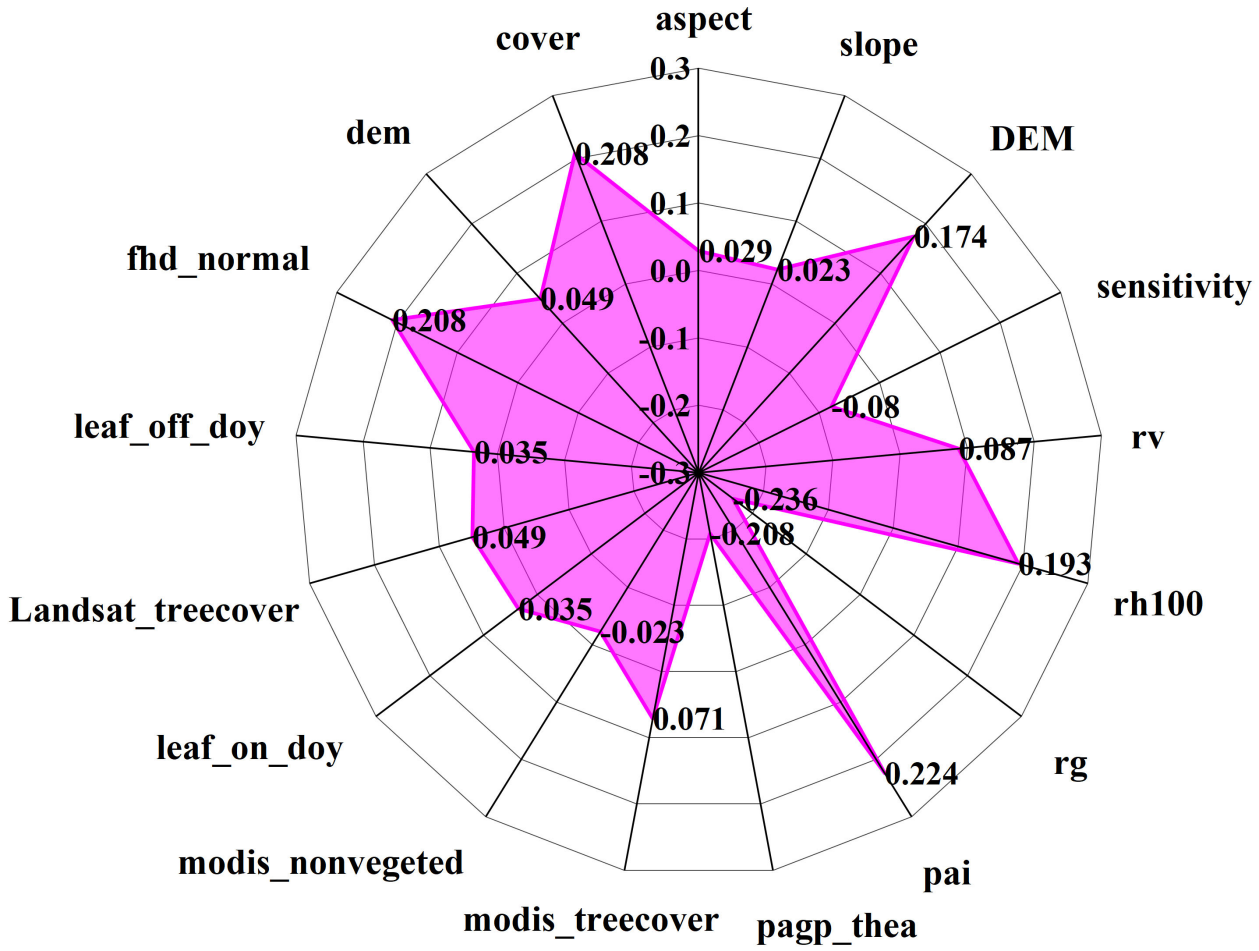
The correlation coefficient between GEDI parameters and AGB of spruce-fir.

Upon examination of **Figure 11**, it is evident that seven characteristic factors of the GEDI independent variables, derived from 100 shots, exhibited significant correlations with the biomass of the plot. These factors, in descending order of correlation strength, are: pai, pgap_thea, cover, fhd_normal, rh100, and DEM. Notably, DEM, rh100, pai, fhd_normal, and cover were found to be significantly positively correlated with AGB, with correlation coefficients falling within the range of 0.174 to 0.224. Conversely, rg and pgap_thea were significantly negatively correlated with AGB, yielding correlation coefficients ranging from -0.208 to -0.236. Notably, among the 14 factors, rg and pai demonstrated the highest correlation, being significantly correlated at the 0.01 level with correlation coefficients of -0.236 and 0.224, respectively. The impact of GEDI L2B provided vegetation leaf area index, leaf height diversity index, and vegetation coverage area on biomass estimation for Shangri-La spruce-fir becomes evident through the analysis of variables. On the other hand, the remaining 10 factors show no significant correlation with the biomass of Shangri-La spruce-fir. Hence, based on the correlation analysis results of each characteristic factor and plot biomass, the top 5 factors with the highest correlation coefficients were selected to serve as the independent variables in the subsequent random forest biomass estimation model.

### 4.6 Biomass modeling

**Figure 12:**
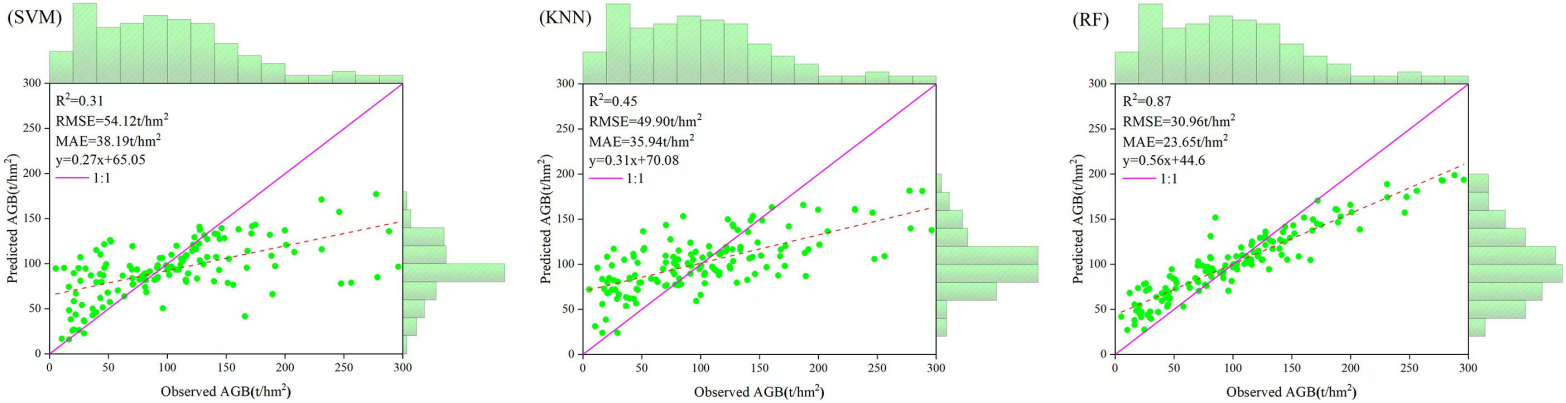
The scatter plots of measured values and predicted values of SVM, KNN and RF models.

**Figure 13:**
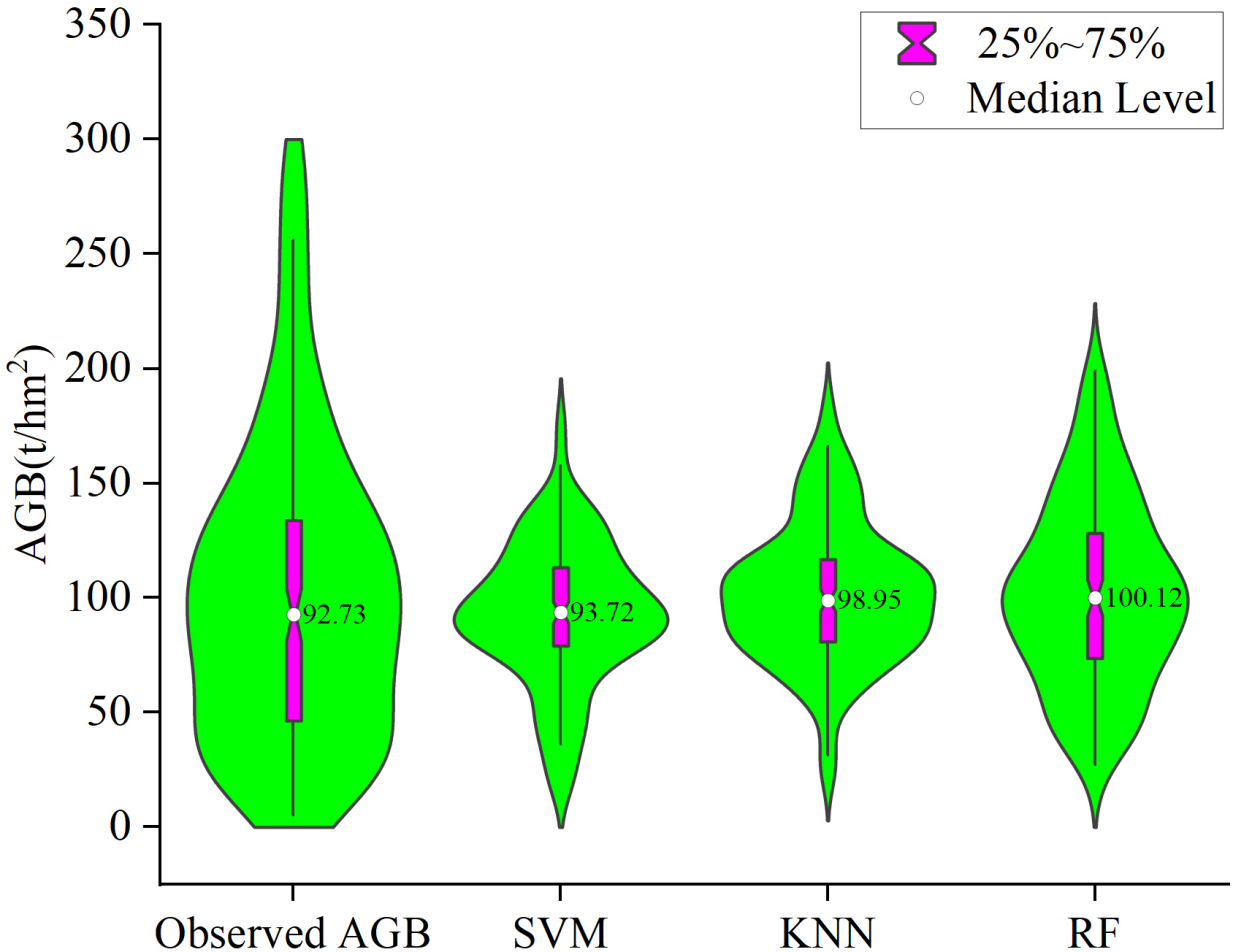
The violin plots of the measured values and predicted values of SVM, KNN and RF models.

In this study, the first five variables with the highest correlation were selected as the modeling factors of support vector machine, k-nearest neighbor method and random forest to estimate the biomass of spruce-fir in the study area, and the accuracy was tested by cross validation. In order to intuitively compare the estimation performance of the three machine learning algorithm estimation models, the relationship between the estimated biomass and the measured biomass was drawn (**Figure 12**). It can be seen from **Figure 3** that the AGB estimation accuracy based on the random forest model is higher, R^2 ^= 0.87, RMSE=30.96t/hm^2^, MAE=23.65t/hm^2^, followed by KNN, R^2 ^= 0.45, RMSE=49.90t/hm^2^, MAE=35.94t/hm^2^, while the SVM model has the lowest estimation accuracy, R^2 ^= 0.31, RMSE=54.12t/hm^2^, MAE=38.19t/hm^2^.From the data distribution map **Figure 13**, it can be seen that the distribution range of AGB predicted values of the three models is 16.19~177.40t/hm^2^, 24.04~181.78t/hm^2^, 27.22~199.10t/hm^2^, respectively, which is less than the measured value range of 5.19~296.10t/hm^2^. Among the three models, random forest has higher estimation accuracy than SVM and KNN, and the overall performance of the estimated value is closer to the measured value, and the model performance is better.

**Figure 14:**
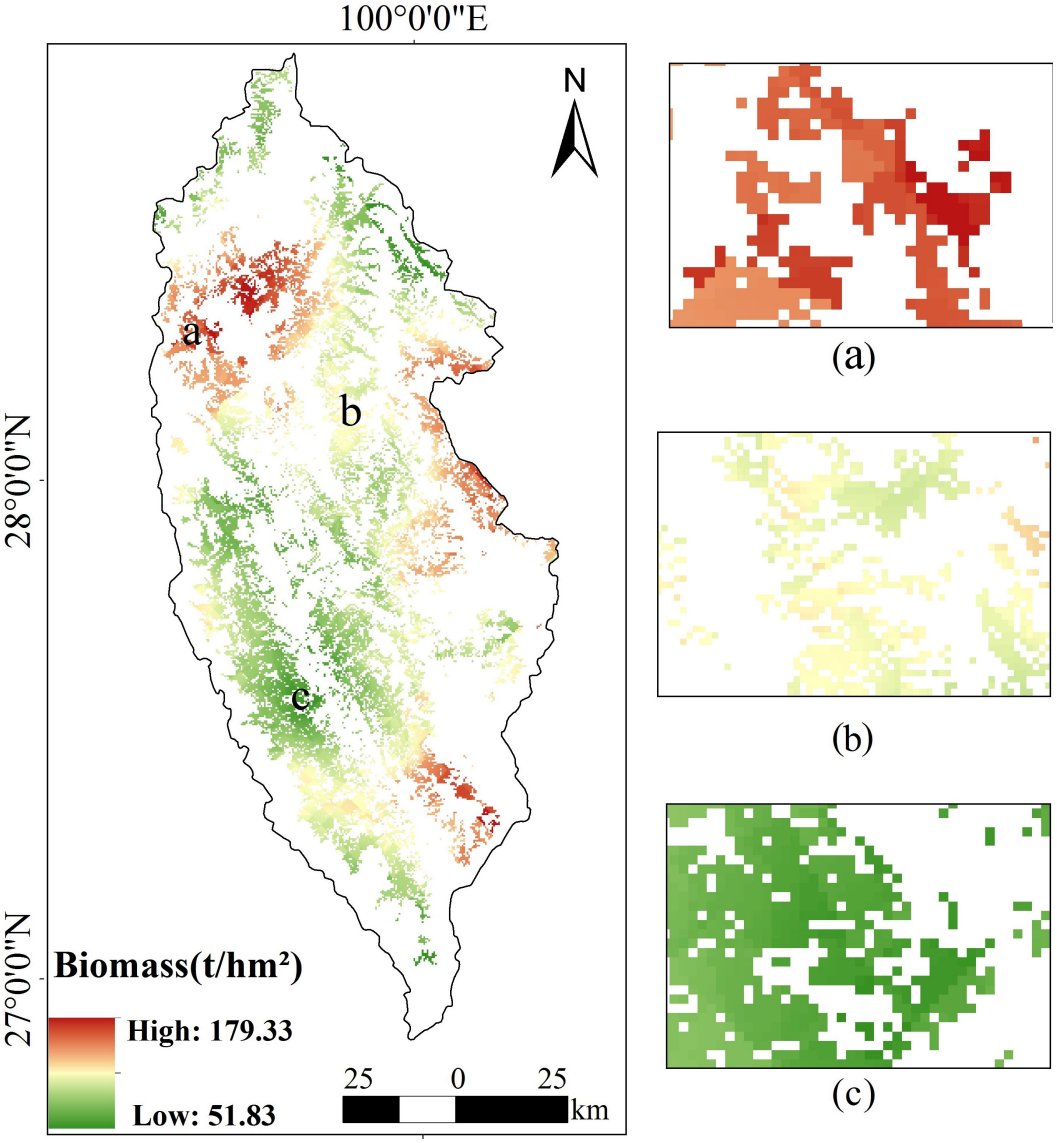
Spatial distribution of aboveground biomass of spruce-fir in Shangri-La, **(A)** high biomass area, **(B)** medium biomass area, **(C)** low biomass area.

Based on the footprints provided by GEDI, the AGB of spruce-fir forest in the whole study area was inverted by using the random forest model with the highest estimation accuracy (**Figure 14**). The random forest estimation revealed that the above-ground biomass of spruce-fir forest in the study area was generally at a medium level, with a maximum of 179.33 t/hm^2^, a minimum of 51.83 t/hm^2^, and an average of 101.98 t/hm^2^. The total biomass value was estimated to be 3035.29×10^4^ t/hm^2^. From the perspective of spatial distribution of biomass, the spruce-fir in the study area showed patches inhomogeneous distribution from northwest to southeast. On the whole, it is distributed longitudinally along the Hengduan Mountains, which conforms to the growth habit of spruce-fir. The biomass of spruce-fir in the northwest and east of Shangri-La is relatively higher than that in the southwest and northeast of Shangri-La. The total biomass of mountain and hilly areas mountainous and hilly areas such as Shangri-La Grand Canyon and Pudacuo National Park display the highest total biomass (**Figure 14A**), while Xiaozhongdian and Shangri-La urban area have the lowest total biomass (**Figure 14C**).

## 5 Discussions

### 5.1 Effects of the distribution and number of GEDI footprint points on the interpolation results and biomass estimation

GEDI is the first space-borne LiDAR satellite dedicated to detecting the three-dimensional structure of vegetation. The beam emitted by GEDI can accurately obtain the vertical structure of vegetation. Its data record the energy returned by different trees at different heights on the ground, including structural information such as surface topography and canopy height, coverage and vertical profile indicators. Most of the previous studies on biomass estimation by GEDI are combined with multi-source remote sensing data extrapolation to obtain continuous forest biomass. For example, Chen et al. (2023) first estimated the biomass on the GEDI footprint point through random forest, and then combined with the characteristic variables of Landsat 8 and Sentinel-1 to estimate the biomass of the entire study area. The ALS biomass extracted by Dorado-Roda et al. (2021) in the GEDI lens is used as an independent variable. The GEDI footprint contains a number of different echo indicators for subsequent GEDI data feature extraction and forest biomass model input. The ALS-derived biomass model is used to estimate the AGB of different forest ecosystems at the GEDI footprint level.

GEDI data are composed of discrete footprint points. After visualization, it can be seen that its distribution law is that the beam emitted along it is evenly distributed on the running track at a distance of 60 m. In our research, different echo indexes of footprints are extended to the surface by using interpolation method. Therefore, in order to verify the effect of interpolation of footprints with different amount and make the distribution of it random and uniform, representative footprints are extracted every 10, 30, 50, 70 and 100 shot when extracting GEDI data. The analysis shows that the interpolation results of the footprint (1309 footprints) extracted every 100 shots are smoother than the other five results (the number of footprints is 13127, 4343, 2646, 1871, respectively). The fewer footprints there are the better the interpolation effect and the higher the prediction accuracy. It can be seen that the distribution and number of GEDI footprints will affect the spatial interpolation results, and also affect the subsequent biomass estimation. Previous studies have shown that the modeling sample size has a significant impact on the uncertainty of the estimation of tree aboveground biomass at the regional scale. Fu et al. (2015) found that the influence of parameter variability of individual tree biomass estimation model caused by modeling sample size on the uncertainty of regional-scale tree aboveground biomass estimation increased with the decrease of modeling sample size, which eventually led to the increase of total uncertainty. Increasing the amount of modeling data can effectively improve the estimation accuracy, accuracy and efficiency of the biomass estimation model, and reduce the uncertainty. In this research, when dealing with footprint data, Python is used to randomly select its number so that it has randomness and distribution uncertainty, so as to disrupt its aggregation distribution phenomenon. In the follow-up study, we can refer to the sample optimization method proposed by Shu et al. (2022), which combine the variance function in geology with the value coefficient in value engineering, or the second-order and third-order spatial sampling methods to extract different numbers of footprint point data (Duncanson et al., 2019).

### 5.2 The influence of GEDI parameter selection on biomass estimation accuracy

In this study, we extracted 14 features from GEDI data that are highly correlated with the AGB of the Shangri-La spruce-fir, including canopy cover area, leaf area index, waveform return ground energy value, etc. These features are used for subsequent random forests to estimate the explanatory variables of the AGB of the spruce-fir. Through correlation analysis, it was found that among all the variables, the variables with the highest correlation with the biomass of spruce-fir in Shangri-La were the integral of the ground component in the waveform (rg), leaf area index (pai), vegetation gap (pgap_thea), canopy cover (cover), leaf height diversity index (fhd_normal). These variables are related to vegetation structure and topography. Several variables with the highest correlation were input into the random forest model, and a higher inversion accuracy (R^2^ = 0.87, RMSE = 30.96 t/hm^2^, MAE=23.65t/hm^2^) was obtained. And **Figure14** shows that the biomass estimation results (3035.29 × 104 t/hm^2^, 101.98 t/hm^2^) in the study area are similar to the results of Yang et al. (2021b) based on the second survey data of forest resources, using the biomass expansion factor method to calculate the biomass of Shangri-La spruce-fir (total value: 3665.9×104 t/hm^2^, average value 113.02 t/hm^2^), indicating that the inversion algorithm based on the parameters provided by GEDI is feasible and the results are reliable.

The predicted biomass of spruce-fir in this study averaged 101.98 t/hm^2^. The estimated biomass at lower positions generally exceeded that at higher positions. This disparity may be attributed to the inclusion of low shrubs and grasslands in the prediction at the sparsely-covered areas beneath the forest canopy, resulting in higher estimates, while the lower predictions at higher positions could be due to the limited penetration of GEDI spaceborne lidar in dense forest areas. Although the saturation of biomass cannot be completely eliminated, the addition of effective remote sensing features can reduce the impact of signal saturation (Qian et al., 2021). Subsequently, the GEDI data can be combined with other image data with reference to previous research methods to overcome the problem of high-value underestimation or low-value overestimation. For example, Xu et al. (2023b) combined Landsat 9 data and GEDI data to invert the carbon storage of Shangri-La Quercus; Silva et al. (2021) integrated GEDI, ICESat-2 and NISAR data to estimate regional-scale biomass, and obtained high estimation accuracy. In addition, although the increase in the number of independent variables makes the estimated biomass closer to the true value, it also reduces the general validity of the biomass model. Therefore, when constructing biomass models, the theories of other disciplines (ecology, biology, meteorology, etc.) should be cross-referenced, and the balance between statistical standards and practical application requirements should be considered (Tamiminia et al., 2021).

### 5.3 The potential and prospect of interpolation technology combined with machine learning model in using GEDI to predict future AGB on a large scale

The discrete footprint points of GEDI lead to the fact that the acquired data are not continuously distributed like optical remote sensing or SAR data. Therefore, most studies combine GEDI and multi-source remote sensing data for large-scale AGB prediction (Kanmegne Tamga et al., 2022; Rodda et al., 2024). In this study, the GEDI footprint is taken as the research object, and the GEDI footprint points are interpolated to obtain the information in the whole research area. By focusing on exploring the influence of the number of different footprint points, that is, the number of sample locations, on the interpolation results. Using different light spot data, the inverse distance weight method is used to interpolate the parameters in the GEDI footprint. Our research results show that the number of light spots is different, and the accuracy of interpolation results is different. The less the number of interpolation spots is, the more dispersed the distribution is, and the closer the interpolation result is to the actual observation value. The machine learning model can capture the complex nonlinear relationship between AGB and explanatory variables through flexible model structure (Pham et al., 2018). Compared with statistical models, machine learning prediction is usually more accurate, so it has been widely used in forest AGB estimation research (Hopman et al., 2021). In this study, based on the interpolation of GEDI in the early stage, GEDI parameters were used as the dependent variable of the random forest model, and spruce-fir sample sites were used as independent variables. The R^2^ of the model predicted by the random forest model was 0.88, RMSE was 30.96 t/hm^2^, and MAE was 23.65 t/hm^2^. The results show that the model has high accuracy. Previous studies have shown that machine learning methods have great potential and advantages in predicting future forest biomass (Li et al., 2020). For example, Liu et al. (2023c) comprehensively considered the contribution of forest factors, site conditions and meteorological factors in modeling, and used BP-ANN and SVM to establish a prediction model for future forest biomass based on sample plots. In the future research, the GEDI parameters can be continuous based on the interpolation method, so as to be added to the biomass model as independent variables together with stand factors, site conditions and meteorological factors, and then the future biomass changes can be predicted by machine learning methods.

### 5.4 Limitations and prospects

In GEDI observations, the reflection of a portion of the laser pulse from a flat bare land or something other than vegetation may lead to biased predictions of information at the footprint level, such as waveform that intersect with buildings, low clouds, steep slopes, rough terrain, or other topographic features, including vegetation and non-vegetation waveform (Bruening et al., 2023). The presence of steep slopes (with or without vegetation) or non-vegetated objects in the waveform footprint will change the value of the relative height index, and may also lead to geodesic errors. If many such observations are used in the mixed estimation, the estimated results may be very different from the unbiased independent reference data. Although the L2B algorithm has a built-in quality marker, when a waveform clearly does not represent surface conditions (such as clouds over the earth ‘s surface), the GEDI algorithm cannot distinguish between waveform from forest canopy and those containing buildings, low clouds, or non-vegetation terrain features, such as rock outcrops, canyon walls, or steep slopes. Therefore, it is necessary to identify these observations and remove them from the set used in the mixed AGB estimation. In this study, GEDI ‘s three filtering conditions were used to eliminate outliers, but the footprint points at buildings, low clouds and non-vegetated terrains were not considered. Subsequent research can refer to Zhang et al.’s (Zhang et al., 2023) filtering of low-quality observation data that is not suitable for AGBD estimation through improved data filtering and GEDI-FIA fusion AGBD model, so as to accurately estimate AGB on the basis of ensuring the quantity and quality of GEDI footprint.

At the same time, the GEDI user manual mentions that although the second version of GEDI ‘s data has improved its geo-positioning accuracy compared to the first version, there is still a position error of 10.2m. The uncertainty of GEDI’s geographical location means that the GEDI ‘s 25m diameter footprint will move within a range of 10.2m. Occasionally, this leads to GEDI data being obtained from adjacent but spatially disjoint regions of footprint locations reported by GEDI products. Therefore, caution will be taken in the use of GEDI data on canopy layers with spatial heterogeneity, such as small stand or forest land with fragmented distribution, and at the edge of the forest (Roy et al., 2021). However, in this study, the surface information is mainly obtained by interpolation using the attribute information in the GEDI footprint point. The geolocation error has little effect on this study, so it is not corrected for geolocation. However, some researchers have proved that the geolocation error of GEDI has little effect on the estimation results (Adam et al., 2020). Therefore, whether it is necessary to do geolocation correction for GEDI application scenarios is the focus and difficulty of the next research.

## Data Availability

The raw data supporting the conclusions of this article will be made available by the authors, without undue reservation.
